# Evaluation of complexity and deliverability of prostate cancer treatment plans designed with a knowledge‐based VMAT planning technique

**DOI:** 10.1002/acm2.12790

**Published:** 2019-12-09

**Authors:** Phillip D. H. Wall, Jonas D. Fontenot

**Affiliations:** ^1^ Department of Physics and Astronomy Louisiana State University and Agricultural and Mechanical College Baton Rouge LA USA; ^2^ Department of Physics Mary Bird Perkins Cancer Center Baton Rouge LA USA

**Keywords:** knowledge‐based planning, quality assurance outcomes, treatment plan complexity, VMAT

## Abstract

**Purpose:**

Knowledge‐based planning (KBP) techniques have been reported to improve plan quality, efficiency, and consistency in radiation therapy. However, plan complexity and deliverability have not been addressed previously for treatment plans guided by an established in‐house KBP system. The purpose of this work was to assess dosimetric, mechanical, and delivery properties of plans designed with a common KBP method for prostate cases treated via volumetric modulated arc therapy (VMAT).

**Methods:**

Thirty‐one prostate patients previously treated with VMAT were replanned with an in‐house KBP method based on the overlap volume histogram. VMAT plan complexities of the KBP plans and the reference clinical plans were quantified via monitor units, modulation complexity scores, the edge metric, and average leaf motion per degree of gantry rotation. Each set of plans was delivered to the same diode array and agreement between computed and measured dose distributions was evaluated using the gamma index. Varying percent dose‐difference (1–3%) and distance‐to‐agreement (1 mm to 3 mm) thresholds were assessed for gamma analyses.

**Results:**

Knowledge‐based planning (KBP) plans achieved average reductions of 6.4 Gy (*P* < 0.001) and 8.2 Gy (*P* < 0.001) in mean bladder and rectum dose compared to reference plans, while maintaining clinically acceptable target dose. However, KBP plans were significantly more complex than reference plans in each evaluated metric (*P* < 0.001). KBP plans also showed significant reductions (*P* < 0.05) in gamma passing rates at each evaluated criterion compared to reference plans.

**Conclusions:**

While KBP plans had significantly reduced bladder and rectum dose, they were significantly more complex and had significantly worse quality assurance outcomes than reference plans. These results suggest caution should be taken when implementing an in‐house KBP technique.

## 1 INTRODUCTION

Knowledge‐based planning (KBP) has received interest within radiation therapy due to its potential to improve treatment plan quality and planning efficiency compared to traditional inverse planning techniques, which has been described extensively in the literature.^1^, ^2^, ^3^, ^4^, ^5^, ^6^ Our group previously reported that the use of KBP provided average reductions of 7.8 Gy and 9.4 Gy in mean dose of the bladder and rectum, respectively, in prostate cancer patients treated with volumetric modulated arc therapy (VMAT) compared to clinical plans.^7^ Although specific implementations of KBP can vary, each method utilizes patient anatomical and planned dosimetric data from previous patients to guide the planning of new patients. KBP systems can serve as quality control tools for clinics to determine whether a treatment plan can be improved by using historical data. For example, Moore et al. developed a simple dose prediction model — based on the fraction of organs‐at‐risk (OARs) overlapping the target volume — capable of predicting mean parotid dose with a standard deviation of just 10% in head‐and‐neck IMRT plans.^8^ Also, Yuan et al*.* used a stepwise multiple regression model to relate patient anatomy to OAR dose and found 85% of inspected OARs were within 10% error in prostate and head‐and‐neck IMRT plans.^9^ More recently, KBP planning modules have become commercially available and also integrated into fully automated planning systems.^10^, ^11^, ^12^ In their detailed review of previous KBP literature, Ge and Wu found that a majority of studies demonstrate how KBP achieved similar and often improved IMRT plan quality along with reduced planning time and plan quality variation.^6^

A trade‐off between plan quality and complexity has been previously reported for static‐gantry intensity‐modulated radiation therapy (IMRT) treatments.^13^, ^14^ In general, improving the dosimetric quality of an IMRT plan can increase its complexity — as measured by increased monitor units (MUs) and beam modulation — if measures for mitigating complexity are not incorporated during optimization. For example, Craft et al. observed that to maximally spare the spinal cord in a lung fixed‐field IMRT case required approximately 7.5 times the number of MUs compared to the most conformal plan, which they determined via Pareto surfaces.^13^ And while a clinically similar plan with only about 2.5 times the number of MU compared to the most conformal plan was achievable, they found the spinal cord dose quickly increased below that point. Therefore, it is often the case increased complexity is necessary to achieve the clinical planning goals such that complexity mitigation methods negatively impact resulting dose distributions.^13^, ^15^

A consequence of increased plan complexity is the potential for reducing the accuracy of the delivered treatment, whereby the impact of uncertainties in relevant delivery parameters — such as leaf positions of the multileaf collimator (MLC) — are exacerbated by small, irregularly shaped beam apertures and narrow leaf gap widths called for by the treatment plan. For instance, Masi et al*.* found that increased VMAT plan complexity (described by modulation complexity scores, or MCS, and leaf travel) was significantly correlated with lower quality assurance outcomes.^16^ This reduced treatment delivery accuracy can have clinical implications.^16^, ^17^ These consequences have led investigators to explore strategies to quantify and reduce plan complexity without compromising plan quality.^18^, ^19^, ^20^

While the advantages of KBP techniques in treatment planning have been widely described, the impact of their use on plan complexity and deliverability are less well understood. Comparatively few studies have even reported on simple complexity surrogates of KBP plans. Hussein et al. found no significant changes in MU and MCS values of prostate IMRT plans when using a commercial KBP system (RapidPlan, Varian Medical Systems, Palo Alto, CA).^21^ Likewise, Tamura et al*.* found no significant changes in patient‐specific quality assurance outcomes when using this commercial KBP system.^22^ Conversely, Kubo et al. reported significantly increased MU values and higher plan complexity when using the same commercial KBP system as the studies noted previously.^23^ These results suggest that further assessment of the complexity and deliverability of KBP‐guided plans is needed. Therefore, the purpose of this study was to examine differences in dose, complexity, and quality assurance outcomes between reference clinical plans and plans designed with an in‐house KBP system.

## 2 METHODS

### 2.1 Treatment plans

A total of 31 prostate cancer patients previously treated at our institution were used for the treatment planning in this study. Selected patients were originally prescribed a dose to a single planning target volume (PTV) and treated using two coplanar, 6 MV VMAT arcs. The clinical plans were originally created using the current treatment planning system at our institution (TPS; Pinnacle^3^ v9.10, Philips Medical Systems, Fitchburg, WI, USA). For research purposes, reference clinical plans were transferred or reconstructed in a research TPS (RayStation v4.5.1.14, RaySearch Laboratories, Stockholm, Sweden), where our in‐house KBP method was developed. These reference clinical plans were recomputed or reoptimized to approximate the original clinical plans (see Figure S1 in the Supplementary Material).

In addition to these reference clinical plans, a KBP‐guided plan was generated for each of the 31 patients using an in‐house KBP technique. The KBP method, based on overlap volume histograms (OVHs) incorporating fractional OAR volumes only within the treatment fields, was used to generate patient‐specific bladder and rectum dose‐volume predictions at the 10%, 30%, 50%, 65%, and 80% relative volume levels. These dose‐volume predictions were then input to the TPS as planning objectives and a KBP‐guided plan was optimized for each patient. Additional details of our KBP method are described elsewhere,^7^, ^24^ but this OVH‐guided KBP method was used in this study because it can be easily implemented clinically and it has been previously shown to predict achievable OAR dose volumes.^25^, ^26^, ^27^ Moreover, it is useful to investigate these types of in‐house KBP systems for clinics that may not have the resources or ability to acquire commercially available KBP systems.

A unique feature of this KBP method is that in addition to the manually constructed clinical plans, the dose database was also populated with standardized Pareto‐optimal plans that equally weighted sparing of each OAR. When a dose volume was queried for patients with similar in‐field OVHs to the new patient, the lowest dose value among both the clinical and Pareto plans was selected as the new patient’s predicted dose volume. Our previous work showed the knowledge from the Pareto plans often resulted in better achievable dose predictions.^7^ So it is important to emphasize that the predicted dose‐volume objectives from this KBP technique are selected from the lowest dose values among the clinical and Pareto plans available in the database.

It is also important to note that the KBP system is separate from the TPS optimization engine, whereby the KBP algorithm predicts dose‐volume objectives to input into the TPS optimizer. For the KBP‐guided plans, the planner strove to achieve the bladder and rectum dose predictions along with originally prescribed physician goals for the target and remaining OARs. Once clinically acceptable target coverage was achieved, OAR sparing was optimized until either the KBP dose predictions were achieved or target coverage became clinically unacceptable.

Each set of 31 reference clinical plans and KBP plans were planned on the same commercial TPS under the same planning conditions, which included the same planner, machine, maximum leaf motion, dose grid resolution, and control point spacing. The primary reason for reconstructing the reference clinical plans was to account for the variations in these parameters that were used to design the original plans. Keeping these parameters and other complexity mitigation tools constant for the reference plans and KBP plans was desired in order to make the fairest comparison between these two sets of plans. All plans were designed to be delivered on a commercial linear accelerator equipped with a 160‐leaf MLC (Infinity, Elekta AB, Stockholm, Sweden) and were optimized with a maximum leaf motion of 7 mm per degree of gantry rotation (mm/deg), dose grid resolution of 4 mm, and control point spacing of 4°. Both reference and KBP plans were optimized under the same conditions with the same dosimetric endpoints for the target and OARs not including the bladder and rectum. The only optimization differences between reference and KBP plans were the bladder and rectum planning objectives, where reference plans utilized the original clinical goals and KBP plans used the predicted dose volumes from the KBP method as described previously.

The plan quality of the reference plans and the KBP plans were compared qualitatively with dose‐volume histograms (DVHs) and quantitatively with Wilcoxon signed‐rank tests on an array of dose metrics at a significance level of *P* = 0.05.

### 2.2 Plan complexity

The complexities of reference plans and KBP plans were quantified using the total planned MUs, MCS,^16^, ^19^ the edge metric (EM),^18^ and the average MLC leaf motion per degree of gantry rotation (LM). Planned MU values were normalized by the fractional prescription dose to enable the comparison between plans of differing prescriptions. The MCS was originally introduced by McNiven et al. to assess fixed‐field IMRT modulation complexity and was later adapted for VMAT by Masi et al.^16^, ^19^ Briefly, the MCS is a metric ranging from zero (most modulated) to one (least modulated) that incorporates leaf sequence variability and aperture area variability components weighted by their segment contributions. The EM is computed as the segment weighted ratio of in‐field MLC side length and aperture area, which was introduced by Younge et al. to characterize the amount of “edge” in apertures.^18^ The values for the scaling factors used in this work were *C_1_* = 0 and *C_2_* = 1. LM was determined by averaging the change in leaf position per degree of gantry rotation calculated at each control point in the plan over each MLC leaf within the jaws.

These metrics were computed directly from the DICOM RT Plan files using in‐house software.^28^, ^29^ MCS, EM, and LM values of the reference and KBP plans were compared using two‐sided paired t‐tests at a significance level of *P* = 0.05. Since the distribution of differences between reference and KBP plan MUs did not meet the normality assumption of the t‐test, a two‐sided paired Wilcoxon signed‐rank test was used for comparing MUs.

### 2.3 Delivery accuracy

Each of the 31 reference and KBP plans were delivered on the commercial linear accelerator platform for which it was planned (Infinity, Elekta AB, Stockholm, Sweden). Dosimetric measurements were performed using a commercial diode array housed in a water‐equivalent phantom (MapCHECK2 and MapPHAN; Sun Nuclear Corporation, Melbourne, FL, USA). Each set of plans was delivered on three separate occasions in order to reduce the effects of measurement noise and fluctuations. The diode array was calibrated prior to each measurement session to eliminate the influence of daily variations in machine output and detector response. While there are several other dosimeters with their own advantages and disadvantages, such as film or EPID panels, the MapCHECK2 was used in this study primarily to mimic the clinical protocol used at our institution. Additionally, while film and EPID panels provide high spatial resolution for relative measurements, they are not ideal absolute dosimeters. Detector arrays also measure dose at detector locations more accurately than film due to processing and densitometry uncertainties and are easier to use compared to film.^30^

Calculated dose distributions were generated for the measurement geometry and plane by the TPS and were compared to measured data using the gamma index in this study. Introduced by Low et al*.*, the gamma index is used to quantify both the percent dose difference (%DD) and distance‐to‐agreement (DTA) between two dose distributions.^31^ Specifically, the gamma index γ is defined as,

(1) $$\Gamma \left(\vec{r_{e}},\;\vec{r_{r}}\right)=\sqrt{\frac{r^{2}\left(\vec{r_{e}},\;\vec{r_{r}}\right)}{\Delta d^{2}}+\frac{\delta^{2}\left(\vec{r_{e}},\;\vec{r_{r}}\right)}{\Delta D^{2}}}$$

(2) $$\gamma \left(\vec{r_{r}}\right)=\min_{}\left\{\Gamma \left(\vec{r_{e}},\;\vec{r_{r}}\right)\right\}\forall \left\{\vec{r_{e}}\right\}$$

where $r\left(\vec{r_{e}},\;\vec{r_{r}}\right)$ is the distance between the reference and evaluated points, $\delta \left(\vec{r_{e}},\;\vec{r_{r}}\right)$ is the dose difference, and Δd and ΔD are the selected DTA and dose difference criteria, respectively. Values of γ equal to or <1 indicate that the comparison passed with respect to the selected %DD and DTA gamma criteria, whereas values greater than 1 indicates failure. Dose normalization also plays an important role in the dose difference criterion. Two possible normalizations are global and local, where the former normalizes dose to the maximum dose in either dose distribution and the latter normalizes dose to the dose at the local point being evaluated. Typical tolerance limits are set as a percentage of points passing, or gamma passing rate (GPR), the given gamma criteria. Task Group No. 218 (TG‐218) recently recommended tolerance and action limits for GPRs to be greater than or equal to 95% and 90%, respectively, using a %DD/DTA gamma criterion of 3%/2 mm with global normalization.^30^

Percent dose difference and distance‐to‐agreement criteria of 3%/3 mm, 2%/2 mm, and 1%/1 mm with both global and local normalization were used to evaluate the agreement between the dose distributions. The 3%/2 mm global criterion was additionally examined to align with the recent recommendations for universal tolerance and action limits from TG‐218.^30^ Gamma passing rates were computed for each plan using commercial quality assurance software (SNC Patient, Sun Nuclear Corporation, Melbourne, FL, USA), where only points with dose above 10% of the maximum dose were included in the analysis. The built‐in calculated shift software feature was used to account for setup uncertainties. Passing rates of the reference and KBP plans were averaged over the three deliveries and statistically compared using two‐sided paired t‐tests at a significance level of *P* = 0.05.

## 3 RESULTS

### 3.1 Plan quality

**Figure 1:**
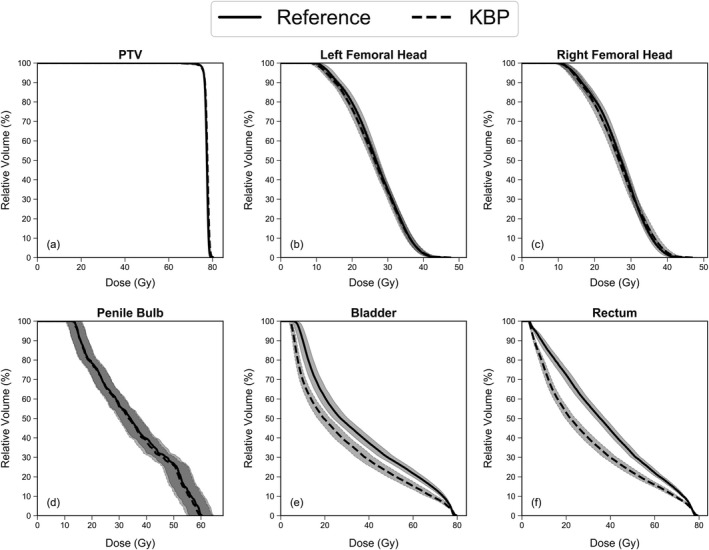
Average DVHs comparing reference clinical plans (solid) and KBP plans (dashed) for the 31 patients of each labeled planning structure (a–f). The standard error of the means is also included as filled bands with solid (reference) or dashed (KBP) edge lines. Note that doses were normalized so that 95% of the PTV received 76 Gy. DVHs, dose‐volume histograms; PTV, planning target volume.

Table 1 shows how reference plans compare statistically with KBP plans for an array of DVH points and dose metrics. While KBP plans showed significant differences in some PTV dose metrics compared to reference plans, it should be noted KBP PTV doses were clinically acceptable and statistically equivalent to the original clinical PTV doses. KBP plans showed significant (*P* < 0.001) decreases in bladder and rectum doses compared to the reference plans. On average, *D*_mean_ for the bladder and rectum was 6.4 Gy and 8.2 Gy lower for KBP plans compared to reference plans, respectively. Average DVHs and the standard errors of the means are shown in Figure 1 for the reference and KBP plans.

**Table 1:** Statistical summary of dose values between reference and KBP plans. Note that all doses were normalized so that 95% of the PTV received 76 Gy.

Dose Metric	Means ± SD		Wilcoxon *p*‐value
	Reference	KBP	Reference vs. KBP
PTV			
*D*_2_ (Gy)	78.7 ± 0.9	79.2 ± 1.1	0.001*
*D*_50_ (Gy)	77.4 ± 0.5	77.7 ± 0.8	0.02*
*D*_98_ (Gy)	75.2 ± 0.9	74.8 ± 0.9	<0.001*
*D*_min_ (Gy)	67.1 ± 6.6	63.3 ± 7.0	<0.001*
*D*_mean_ (Gy)	77.3 ± 0.5	77.6 ± 0.7	0.013*
*D*_max_ (Gy)	79.6 ± 1.3	80.6 ± 1.5	<0.001*
*V*_95_ (%)	99.7 ± 0.7	99.5 ± 0.8	<0.001*
*V*_98_ (%)	99.0 ± 1.2	98.4 ± 1.2	0.77
*V*_100_ (%)	94.6 ± 2.3	94.8 ± 1.4	0.98
*V*_107_ (%)	0.02 ± 0.1	0.2 ± 0.5	0.011*
*HI* †	0.05 ± 0.02	0.06 ± 0.02	<0.001*
*CI* †	1.4 ± 0.1	1.4 ± 0.06	0.002*
Bladder			
*D*_10_ (Gy)	73.6 ± 6.1	68.4 ± 13.8	<0.001*
*D*_30_ (Gy)	48.5 ± 18.7	38.2 ± 22.7	<0.001*
*D*_50_ (Gy)	27.2 ± 18.5	19.5 ± 16.2	<0.001*
*D*_65_ (Gy)	17.9 ± 15.7	11.6 ± 9.7	<0.001*
*D*_80_ (Gy)	12.5 ± 13.0	7.7 ± 6.5	<0.001*
*D*_mean_ (Gy)	35.1 ± 12.8	28.6 ± 12.0	<0.001*
Rectum			
*D*_10_ (Gy)	72.8 ± 4.6	69.1 ± 7.9	<0.001*
*D*_30_ (Gy)	51.4 ± 12.1	39.8 ± 17.0	<0.001*
*D*_50_ (Gy)	35.4 ± 12.8	21.9 ± 12.9	<0.001*
*D*_65_ (Gy)	24.6 ± 12.9	13.8 ± 9.0	<0.001*
*D*_80_ (Gy)	15.0 ± 11.9	8.9 ± 6.5	<0.001*
*D*_mean_ (Gy)	38.0 ± 8.9	29.8 ± 9.3	<0.001*
Left Femoral Head			
*D*_2_ (Gy)	40.1 ± 6.7	40.4 ± 7.1	0.019*
*D*_max_ (Gy)	45.9 ± 10.4	47.8 ± 10.2	<0.001*
*D*_mean_ (Gy)	26.6 ± 5.1	26.1 ± 5.6	0.95
Right Femoral Head			
*D*_2_ (Gy)	39.5 ± 6.8	40.5 ± 7.7	<0.001*
*D*_max_ (Gy)	44.8 ± 9.8	47.0 ± 9.3	<0.001*
*D*_mean_ (Gy)	26.8 ± 5.1	26.6 ± 6.0	0.65
Penile Bulb			
*D*_mean_ (Gy)	35.5 ± 18.4	34.9 ± 18.9	0.18

PTV, planning target volume.

* Indicates a statistically significant result of *P* < 0.05.

^†^ Homogeneity and conformity indices were calculated according to their International Commission on Radiation Units & Measurements definitions.

### 3.2 Plan complexity

KBP plans were significantly more complex than reference plans in every evaluated metric. On average, KBP plans required 143 more MUs (*P* < 0.001), had reduced MCS values of 18% (*P* < 0.001; indicating increased complexity), had 40% higher EM values (*P* < 0.001), and 47% higher LM (*P* < 0.001) compared to reference plans (Table 2).

**Table 2:** Statistical summary of the differences in complexity metrics between the reference and KBP plans

Complexity Metric	Reference Plans (μ ± σ)	KBP Re‐plans (μ ± σ)	t‐test *p*‐value
MU	450 ± 83	593 ± 113	<0.001*†
MCS	0.5 ± 0.1	0.4 ± 0.1	<0.001*
EM	0.06 ± 0.02	0.08 ± 0.01	<0.001*
LM (mm/deg)	1.0 ± 0.6	1.5 ± 0.5	<0.001*

EM, edge metric; LM, leaf motion; MCS, modulation complexity scores; MUs, monitor units.

* Indicates a statistically significant result of *P* < 0.05.

^†^ Result from two‐sided Wilcoxon signed‐rank test because the distribution of differences in MUs between reference and KBP plans was determined to break the t‐test assumption of normality.

Complexity metrics were shown to be strongly correlated with each other. An increase in MUs correlated with more complex MCS, EM, and LM values with Pearson correlation coefficients (R) of −0.85 (*P* < 0.001), 0.91 (*P* < 0.001), and 0.84 (*P* < 0.001), respectively. Similarly, an increase in complexity in terms of the MCS score correlated strongly with an increase in EM values (R = −0.94; *P* < 0.001) and LM (R = −0.88; *P* < 0.001). Lastly, an increase in EM values corresponded strongly with more LM with R = 0.85 (*P* < 0.001).

### 3.3 Delivery accuracy

Knowledge‐based planning plans showed significant reductions in quality assurance outcomes compared to reference plans as described by GPRs. For criteria with global normalization, KBP plans on average had GPRs that were 1.1, 1.6, 3.8, and 7.8 percentage points lower than reference plans at the 3%/3 mm (*P* = 0.009), 3%/2 mm (*P* = 0.003), 2%/2 mm (*P* = 0.002), and 1%/1 mm (*P* < 0.001) criteria, respectively. Significant reductions in KBP plan GPRs compared to the reference plans were also observed at each evaluated gamma criteria using local normalization (Table 3). Additionally, it is notable that KBP plans showed significantly greater inter‐delivery variations (*P* < 0.05) in GPRs than reference plans at each gamma criteria for both global and normalization methods (see Table S1).

**Table 3:** Statistical summary of the differences in gamma passing rates between the reference and KBP plans at different gamma criteria.

	Gamma Criteria	Reference Plans Gamma Pass Rates (μ ± σ)	KBP Plans Gamma Pass Rates (μ ± σ)	t‐test *P*‐value
Global	3%/3 mm	98.8 ± 1.3	97.7 ± 2.5	0.009*
	3%/2 mm	98.3 ± 1.7	96.6 ± 3.3	0.003*
	2%/2 mm	93.8 ± 4.2	90.0 ± 6.8	0.002*
	1%/1 mm	69.7 ± 8.7	61.9 ± 11.7	<0.001*
Local	3%/3 mm	91.8 ± 4.4	88.9 ± 6.6	0.03*
	2%/2 mm	87.4 ± 6.0	82.0 ± 9.5	0.003*
	1%/1 mm	75.4 ± 8.7	66.5 ± 11.9	<0.001*

* Indicates a statistically significant result of *P* < 0.05.

Patient‐specific QA outcomes at the 3%/3 mm global gamma criterion for all reference plans were greater than 95%. As for the KBP plans, only two plans had passing rates of less than 95% but greater than 90%. One KBP plan had a GPR of lower than 90% (87.7%).

Gamma passing rates were also found to be weakly to moderately correlated with the evaluated plan complexity metrics (Table 4). For instance, GPRs at the 2%/2 mm local criterion moderately correlated with MUs (R = −0.47; *P* < 0.001), MCS values (R = 0.42; *P* < 0.001), EM values (R = −0.40; *P* = 0.001), and LM (R = −0.37; *P* = 0.003).

**Table 4:** Pearson correlation coefficients between complexity metrics and gamma passing rates.

	Pearson Correlation Coefficients (*P*‐value)				
	Gamma Criteria	MU	MCS	EM	LM
Global	3%/3 mm	−0.37 (0.003*)	0.36 (0.004*)	−0.36 (0.004*)	−0.33 (0.009*)
	3%/2 mm	−0.43 (< 0.001*)	0.40 (0.001*)	−0.40 (0.001*)	−0.38 (0.002*)
	2%/2 mm	−0.45 (< 0.001*)	0.39 (0.002*)	−0.39 (0.002*)	−0.36 (0.005*)
	1%/1 mm	−0.50 (< 0.001*)	0.45 (< 0.001*)	−0.46 (< 0.001*)	−0.40 (0.001*)
Local	3%/3 mm	−0.35 (0.006*)	0.31 (0.02*)	−0.29 (0.02*)	−0.21 (0.10)
	2%/2 mm	−0.47 (< 0.001*)	0.42 (< 0.001*)	−0.40 (0.001*)	−0.37 (0.003*)
	1%/1 mm	−0.56 (< 0.001*)	0.50 (< 0.001*)	−0.50 (< 0.001*)	−0.49 (< 0.001*)

EM, edge metric; LM, leaf motion; MCS, modulation complexity scores; MUs, monitor units.

* Indicates a statistically significant result of *P* < 0.05

## 4 DISCUSSION

In this work, VMAT plans for prostate cancer patients designed with an OVH‐guided KBP method were significantly more complex and had significantly lower patient‐specific quality assurance outcomes compared to manually constructed reference plans. While KBP‐guided plans led to significant improvements in OAR sparing, the values of MU, MCS, EM, and LM were all significantly more complex. In addition, a weak to moderate correlation was observed between the analyzed complexity metrics and quality assurance outcomes.

To our knowledge, this work is the first evaluation of plan complexity and deliverability of plans derived from an *OVH‐guided* KBP method, whereas other studies have reported results from the commercial KBP product*, RapidPlan*. The observed improvements in KBP plan quality are consistent with previous studies investigating KBP methods for prostate cancer.^32^, ^33^ The OAR dose‐volume predictions generated from the OVH‐guided KBP model are designed to output the lowest achievable dose levels based on previous data. The results from this study indicate that the achievability of these predictions seem to come at the cost of significant increases in plan complexity, which is consistent with the work of Kubo et al.^23^ On the other hand, Tamura et al. reported KBP plans to have similar complexity to reference plans overall. They also observed significantly reduced (*P* < 0.05) leaf travel in KBP plans. Both Tamura et al*.* and Kubo et al. each evaluated 30 prostate patients using the same commercial KBP system. It is worth highlighting the differences between the KBP method used in the present study and the commercial system (RapidPlan) used in these previous studies. While the RapidPlan training algorithm uses model‐based principal component regression,^10^ the KBP system in this work follows an established library lookup algorithm to find the lowest dose achieved among a database of previously treated patients with similar in‐field OVHs to the new patient.^26^ Also, as mentioned previously in Section 3, standardized Pareto‐optimal plans were added to the dose database and were found to further improve OAR sparing overall compared to using data from manually constructed clinical plans.^7^ This distinguishes this OVH‐driven KBP system from RapidPlan’s regression‐model technique. Therefore, one possible explanation for this discrepancy among previous works could be the differences in dose objectives and resulting distributions*, *that is, the extent to which one study more aggressively pursued a better plan result than the other. Furthermore, the differences between previous findings and our results may be explained by the differing KBP techniques and also differences in the quality of the underlying dose databases these KBP systems used to generate their dose objectives. While KBP plans in both previous studies achieved similar dose to clinical plans overall, KBP plans reported by Kubo et al. showed significantly lower mean bladder dose along with significantly higher MUs and more complex MCS values. Our study achieved similar bladder dose reductions as Kubo et al. These results therefore suggest the possibility that increased complexity may be required in order to meet the “ideal” dose objectives, in which case efforts to mitigate complexity may reduce the quality of the KBP‐guided dose distributions.

We did not observe a strong correlation between improved bladder mean dose and increased complexity and only a moderate (R ≥ 0.48) correlation between improved rectum mean dose and increased complexity (see Figure S4 in the Supplementary Material). Plan complexity metrics were also not strongly correlated with GPRs. This observation is consistent with previous works^16^, ^34^ and could indicate the selected plan quality metrics cannot fully describe plan complexity, even though available evidence suggests a relationship does exist. While there have been studies showing increased monitor units are necessary for achieving desired dose distributions for certain IMRT cases with complex geometry,^13^ other studies have observed instances of unnecessary VMAT plan complexity and were able to reduce complexity without substantially impacting plan quality using complexity penalties.^18^ As Mohan et al. noted, the amount of possible complexity reduction is dependent on the difficulty of the underlying treatment plan.^13^, ^15^ However, it remains uncertain whether the increased complexity observed in the KBP plans of this study was required for the improved OAR dose. Further investigation into what extent the complexity of these KBP plans could be mitigated by exploring different TPS optimization settings is warranted.

The clinical implications of increased plan complexity and reduced delivery accuracy have been studied extensively, which served as a primary motivation for this study. Investigators such as Younge et al. have implemented aperture complexity penalties into the plan optimization stage to limit plan complexity without degrading plan quality.^18^ Others have examined how an array of metrics that quantify beam complexity (such as leaf travel in addition to plan irregularity and modulation) correlate with delivery accuracy and pretreatment verification results.^17^, ^20^ Valdes *et al*. recently showed the feasibility of using machine learning techniques to accurately predict GPRs of IMRT plans using many complexity features.^35^, ^36^ It is possible that further accounting for plan complexity using these similar methods during the optimization stage could reduce KBP’s observed impact on complexity on a plan‐specific level, thereby providing a more accurate delivery. This is an avenue of research we plan on investigating in future work.

This study had several limitations. Planning time was not explicitly recorded in this research since KBP has been extensively shown to improve planning efficiency.^1^, ^37^, ^38^, ^39^ However, average KBP planning time was qualitatively comparable to average reference planning time in the present study. Also, standard clinical values for control point spacing and dose grid resolution were used in this work. While it is possible increasing the resolution of these two parameters could mitigate the observed differences in calculated and measured KBP dose,^16^ the settings used in this study have been shown to provide an acceptable balance of calculation accuracy and speed.^40^, ^41^ Also, the leaf motion was not constrained beyond the default limits of the modeled linear accelerator. It is possible that adjusting these specific optimization parameters may diminish KBP plan complexity and delivery accuracy deficiencies to an extent.^42^, ^43^, ^44^ Another potential limitation is that only a limited number of metrics were chosen to quantify plan complexity, though the chosen metrics are commonly used in the literature.^16^, ^20^

The use of a diode array also presents potential limitations. A previous study has observed a slight temperature dependence for individual MapCHECK diodes ranging from 0.52% to 0.57%/°C.^45^ The impact of any existing temperature dependence would likely be negligible in the present study as the measurements for KBP and reference plans were acquired consecutively and in temperature‐controlled rooms. Also, other studies have shown an angular dependence to be the factor that most affects the accuracy of MapCHECK2 measurements — particularly at gantry angles of 90 and 270° — which could potentially affect GPRs.^46^, ^47^ While this study did not directly investigate the effects on these temperature and angular dependencies on delivery accuracy, the gamma passing rates at clinically relevant criterion for the plans in this study were consistent with those observed at our clinic for prostate cases. Additionally, the commercial diode array used in this work has been shown to provide accurate VMAT QA measurements despite this angular dependence.^47^, ^48^, ^49^ It is also important to note the results seen here with this specific combination of technologies (OVH‐guided KBP method, RayStation TPS, Elekta treatment machine, and MapCHECK2 diode array) may not hold for different KBP methods and planning, delivery, and measurement technologies as evidenced by the results from Tamura et al.^22^ Regardless, the results of this study indicate that caution is needed regarding the effects of plan complexity and quality assurance outcomes when implementing any KBP system as they become more clinically prevalent. However, these results supplement the available literature showing KBP’s potential in providing immediate and substantial clinical impact. In‐house OVH‐guided KBP systems similar to the one described in this work could be developed and implemented clinically without disrupting the existing inverse optimization workflow. The KBP system would provide patient‐specific predicted bladder and rectum dose‐volume planning objectives prior to planning, and the planner could then strive to meet these KBP goals as they would normally. The observed increase in complexity and reduction in QA outcomes from this study may warrant additional focus on the quality control of KBP plan delivery. The qualified medical physicist would be responsible for monitoring the deliverability of VMAT plans designed with any KBP system. Provided that any reduction in QA outcomes does not result in consistently unacceptable plans, the substantial potential improvement in plan quality provided by KBP systems should persuade the clinical physicist to investigate the feasibility of implementing a KBP system within his or her institution.

Our study did not investigate the source of the reduced quality assurance outcomes of KBP‐guided plans. While it would certainly be important and desirable to characterize the specific causes of delivery accuracy discrepancies between KBP and reference plans we leave this for future research as it lies outside the scope and purpose of the current work. However, there are known categories of uncertainties in the IMRT planning and delivery process that include: limitations of the beam model (e.g., MLC modeling, modeling output factors for small fields, etc.), mechanical and dosimetric uncertainties of the delivery system (e.g., MLC leaf position and speed errors, gantry rotation and table motion stability, beam stability, etc.), and measurement and analysis uncertainties (e.g., setup errors).^30^ Given evidence available in the literature, the primary source of error in the discrepancies between KBP and reference GPRs is most likely inaccuracies in the TPS dose computation. For instance, Masi et al. observed increased GPRs with plans optimized with a finer control point spacing compared to plans of similar complexity optimized with a courser control point spacing.^16^ Therefore, since the KBP and reference plans were optimized under the same TPS settings, the resulting differences in GPRs may primarily be caused by limitations in the TPS’s ability to accurately model and compute dose of plans of higher complexity. Increasing the control point spacing resolution during KBP plan optimization could mitigate the observed delivery errors to some extent, but future work is needed to fully describe the specific sources of error. KBP effects on plan complexity and GPRs for different treatment sites, such as the head and neck, would also be instructive to explore. But overall, this research gives reason to further validate and verify all aspects of the treatment workflow when implementing KBP systems, whether they be established in‐house or commercially available methods.

## 5 CONCLUSION

While KBP methods have been shown to improve the quality and consistency of treatment plans across institutions, the results of this study suggest their use can significantly increase plan complexity and reduce patient‐specific QA outcomes. An in‐field OVH‐guided KBP method was used to generate 31 VMAT plans for previous prostate cancer patients. KBP plans showed significantly reduced bladder and rectum dose but were significantly more complex compared to reference plans. The KBP plans showed a significant reduction in delivery accuracy — as measured by patient‐specific QA measurements. These results demonstrate that care should be taken when implementing KBP models to ensure resulting plans achieve acceptable quality and deliverability.

## CONFLICT OF INTEREST

The authors have no conflicts of interest to disclose.
