# Supplementary material for: Evaluation of complexity and deliverability of prostate cancer treatment plans designed with a knowledge‐based VMAT planning technique
Source: J Appl Clin Med Phys. 2019 Dec 9;21(1):69–77. doi: 10.1002/acm2.12790 (PMC6964749; doi:10.1002/acm2.12790)
Supplement: Supplementary file 1 [file ACM2-21-69-s001.pdf]

Table S1: Statistical summary of the differences in coefficients of variation (COV) of inter-delivery measurements at each gamma criteria between reference and KBP plans over the three separate measurements.

	Gamma Criteria	Reference Plans COV ($\mu \pm \sigma$ x 10^{-2})	KBP Plans COV ($\mu \pm \sigma$ x 10^{-2})	t-test *p*-value
Global	3%/3mm	0.3 ± 0.3	0.5 ± 0.6	0.005*
	3%/2mm	0.4 ± 0.4	0.8 ± 0.7	0.001*
	2%/2mm	0.8 ± 0.5	1.8 ± 1.4	< 0.001*
	1%/1mm	3.1 ± 2.1	4.8 ± 3.4	0.005*
Local	3%/3mm	0.9 ± 0.6	1.3 ± 1.0	0.02*
	2%/2mm	1.3 ± 1.0	2.1 ± 1.6	0.004*
	1%/1mm	2.8 ± 1.9	4.2 ± 2.4	0.003*

*Indicates a statistically significant result of $p < 0.05$

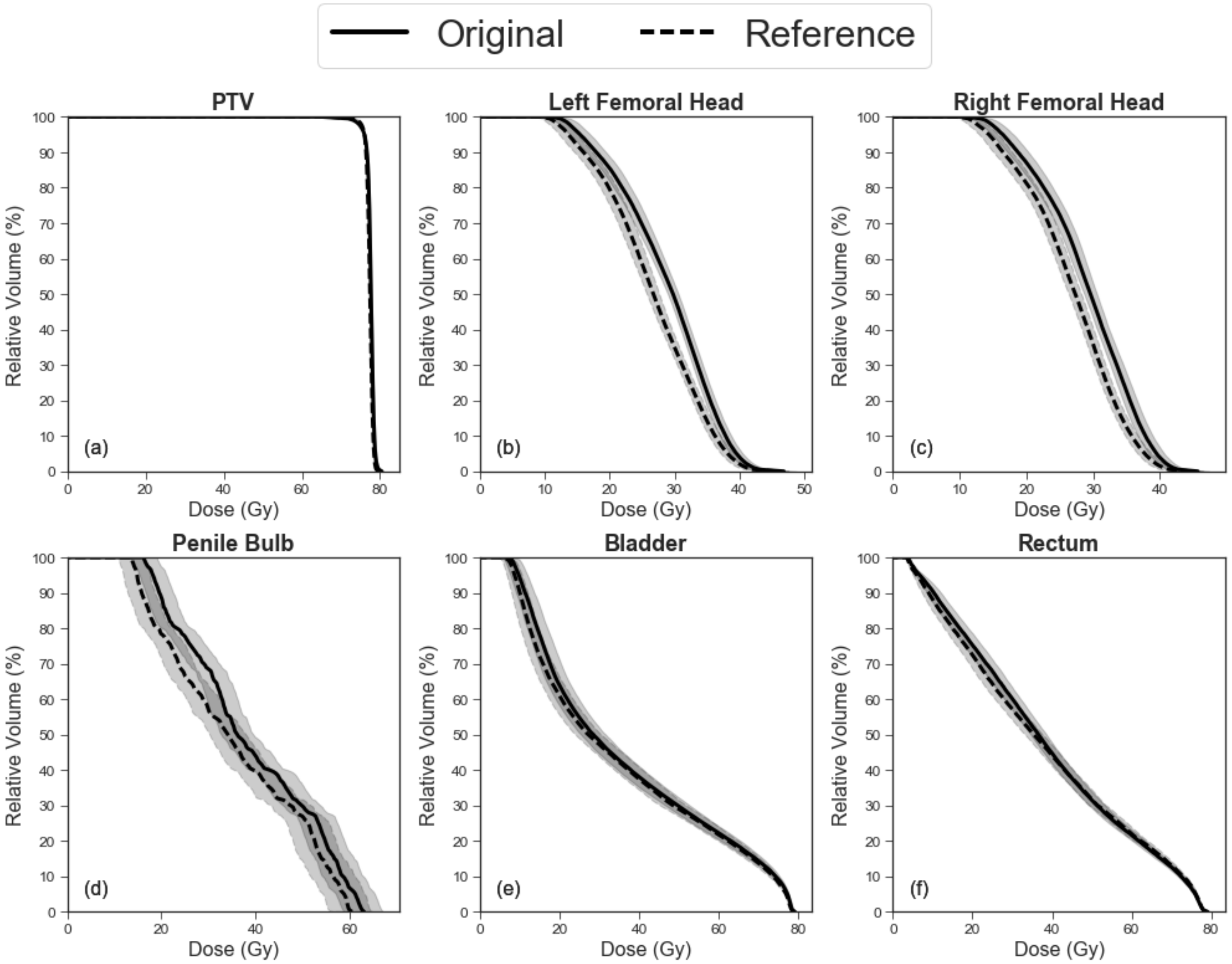

Figure S1: Average DVHs comparing original clinical plans (solid) to the reconstructed reference clinical plans (dashed) for the 31 patients of each labelled planning structure (a-f). The standard error of the means is also included as filled bands with solid (original) or dashed (reference) edge lines. Note that doses were normalized so that 95% of the PTV received 76 Gy.

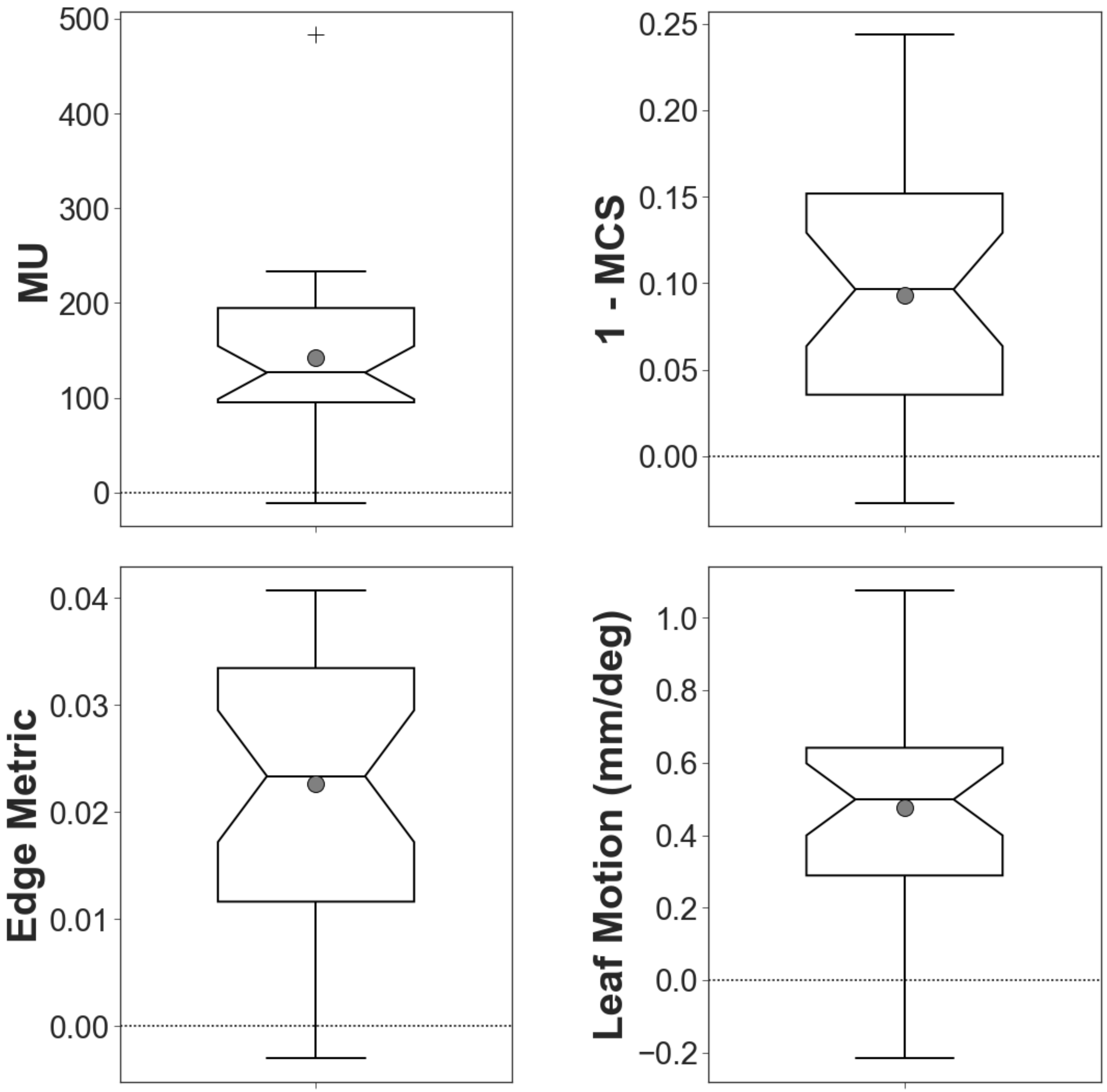

Figure S2: Distributions of the 31 paired differences between KBP and reference plans for planned MUs (a), MCS values (b), EM values (c), and LM (d). Positive values in each complexity metric plot indicate the KBP value was larger (i.e. more complex) than the corresponding reference plan value. Note in (b), 1 – MCS values were plotted so that higher values indicate higher complexity in each plot. Horizontal black lines within each box indicate distribution medians; notches indicate the 95% confidence intervals around each median, grey circles indicate the distribution means; whiskers indicate the range of data points lying within the 1.5 times the interquartile range and crosses indicate points outside this range.

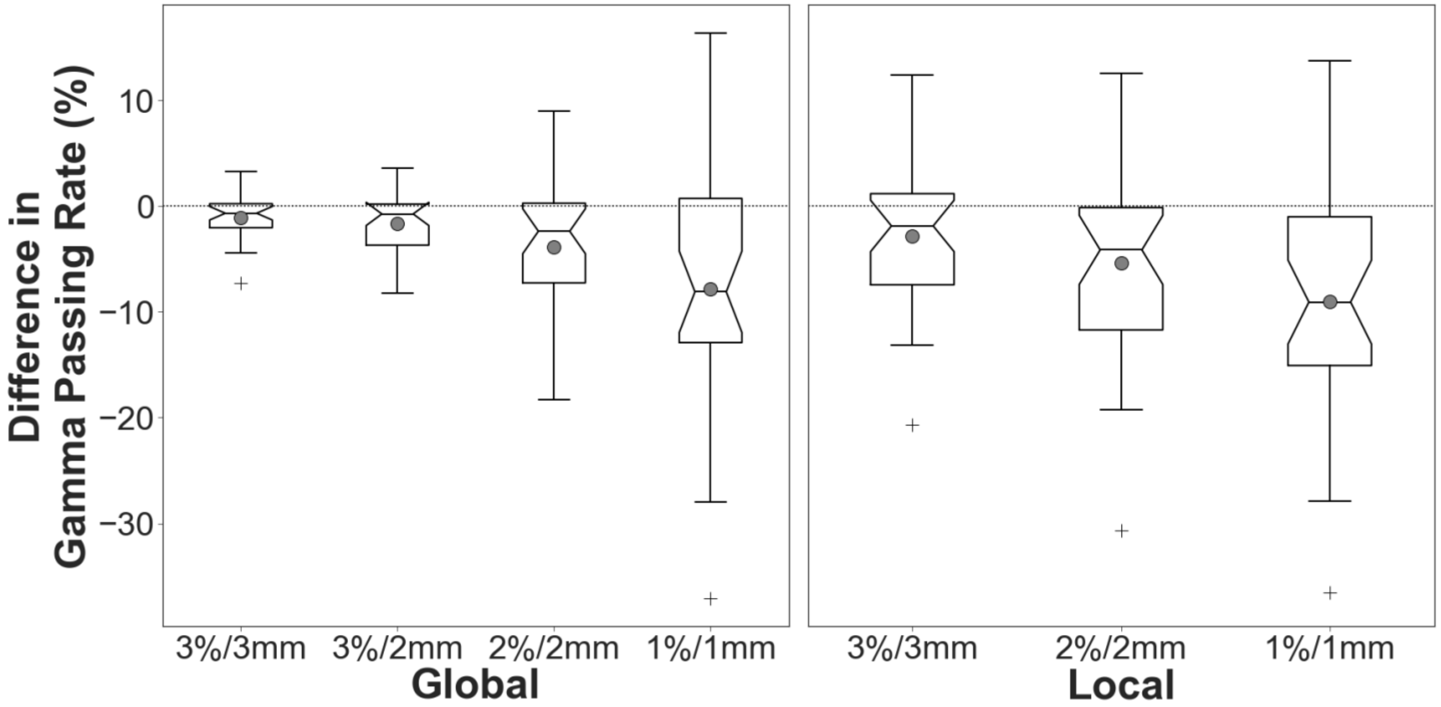

Figure S3: Distributions of differences in gamma passing rates between reference plans and KBP plans at each gamma index criteria calculated with both global (left) and local (right) normalization. Negative values indicate the KBP plan had a lower gamma passing rate. Same boxplot characteristics from the Figure S2 caption apply here.

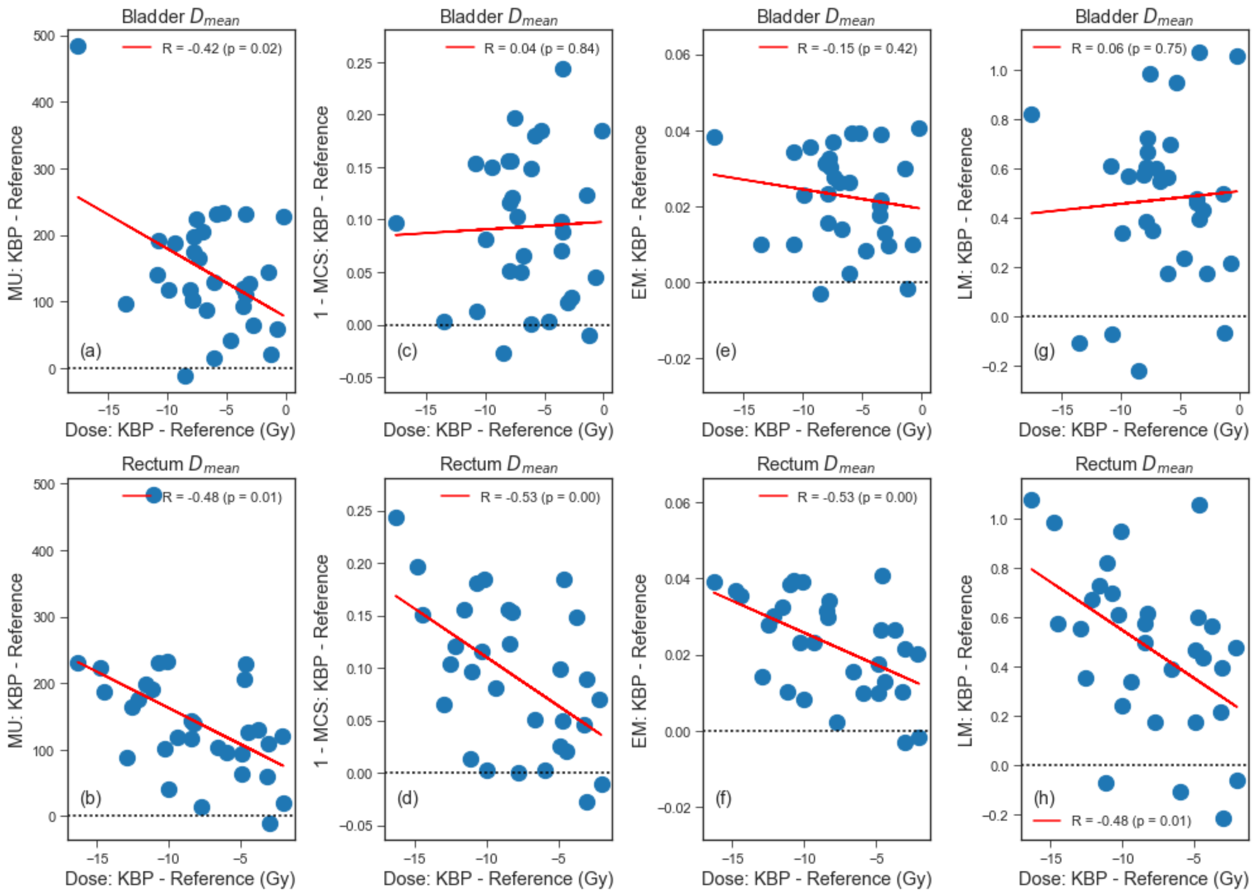

Figure S4: Correlation between increased plan complexity and improvement in plan quality. Differences between KBP and reference plans are shown, where positive y values indicate increased KBP plan complexity and negative x values indicate improved or lower bladder (a, c, e, g) or rectum dose (b, d, f, h).
